# Both-Bone Forearm Shaft Fractures Treated with Compression Plate Fixation in Adults: A Systematic Review on Adverse Events and Outcomes

**DOI:** 10.2106/JBJS.OA.24.00129

**Published:** 2024-11-26

**Authors:** Henri Vasara, Antti Stenroos, Samuli Aspinen, Jussi Kosola, Turkka Anttila, Panu H. Nordback

**Affiliations:** 1 Department of Surgery, Central Finland Hospital Nova, Jyväskylä, Finland; 2 University of Helsinki, Helsinki, Finland; 3 Department of Orthopedics and Traumatology, Hyvinkää Hospital, Hyvinkää, Finland; 4 Department of Hand Surgery, Helsinki University Hospital, Helsinki, Finland

## Abstract

**Background:**

Both-bone forearm shaft fractures (BBFFs) in adults carry a significant risk of adverse events (AEs). Based on the current literature, there is considerable variance in AE incidence reporting. We aimed to systematically review the literature on BBFFs in adults treated with compression plate fixation, assessing AEs and long-term outcomes.

**Methods:**

We performed a systematic review based on the PubMed database on the current literature on adult BBFFs treated with open reduction and internal fixation with compression plates. Two authors independently collected the data, and a third author resolved disagreements between the 2 reviewers. The primary outcome measure was postoperative AEs, whereas the secondary outcome was to review the long-term outcomes. We evaluated the methodological quality of the studies with a modified version of the Coleman Methodology Score.

**Results:**

Fifteen studies (12 retrospective case series and 3 randomized controlled trials) met the set inclusion criteria. In total, there were 944 patients, of whom 24% (n = 224) experienced some AEs, and 14% had major AEs requiring secondary operations or remaining persistent. The most common AEs were postoperative nerve injuries (incidence 7%, n = 64/944) and fracture nonunion (incidence 5%, n = 45/944). Disabilities of the Arm, Shoulder, and Hand scores were available for 135 patients (5 studies), with a mean score of 12.5 (range 0-61). According to the modified Coleman Methodology Scores, there were 2 good-, 1 fair-, and 12 poor-quality studies among the included studies.

**Conclusion:**

BBFF compression plate fixation in adults poses a relatively high AE risk (24%). According to available patient-reported outcomes and range of motion measurements, the average postoperative outcomes are good, although a minor disability typically persists to some extent. There is a need for high-quality prospective trials assessing the treatment and outcomes of BBFFs in adults to improve forearm fracture treatment.

**Level of Evidence:**

Level III. See Instructions for Authors for a complete description of levels of evidence.

## Introduction

Both-bone forearm shaft fractures (BBFFs) are relatively rare high-energy injuries most often sustained by young working-age males with high functional demands^1-3^. These fractures are unstable being prone to primary or progressive dislocation with conservative treatment. Therefore, the standard treatment in adults is operative if not explicitly contraindicated^4,5^. Open reduction and internal fixation with compression plates is currently the preferred method of fixation, although intramedullary fixation of both or either bone has been proposed^6,7^. Successful treatment is crucial as forearm function substantially affects performance in daily activities^8^. After adequate reduction and fixation, the functional outcomes are usually satisfactory^9^. However, only a few studies report functional and patient-reported outcomes after BBFF fixation with varying results^10-12^.

The most used compression plates are the locking compression plate (LCP)^13^ and limited-contact dynamic compression plate (LC-DCP). In addition, older-generation dynamic compression plates (DCPs) are also still used. LCPs and LC-DCPs have limited underlying implant surfaces facing the bone and, therefore, possess a superior fracture union rate compared with the DCP^14^. Tubular or reconstruction plates should be avoided as they have a higher incidence of nonunion and other adverse events (AEs)^2,4^. Otherwise, there is no evidence regarding the superiority of the available compression plate models^15^.

BBFFs in adults carry a significant risk of AEs. Most publications report incidences between 20% and 40% depending on the inclusion criteria^2,6,16-18^. However, there is considerable inconsistency in AE incidences as the highest reported incidence is 45%^3^, whereas the lowest is 5%^19^. The most common AEs include nonunion, nerve injuries, and surgical site infections.

Currently, there are no systematic reviews conducted on the AEs after BBFFs with compression plating in adults. Thus, our aim was to perform a systematic literature review on BBFFs in adults treated with open reduction and compression plate fixation. Our primary aim was to establish the incidence of different AEs after operatively treated BBFFs. Our secondary aim was to evaluate the patient-reported outcomes, functional outcomes, range of motion, and grip strength.

## Materials and Methods

We performed a systematic review of the current literature on adult BBFFs treated with open reduction and internal fixation with compression plates. We used the Preferred Reporting Items for Reporting Systematic Meta-Analyses 2020 Guidelines during the review process^20^. We registered the review in the International Prospective Register of Systematic Reviews PROSPERO (CRD42024510151).

### Literature Search

**Fig. 1:**
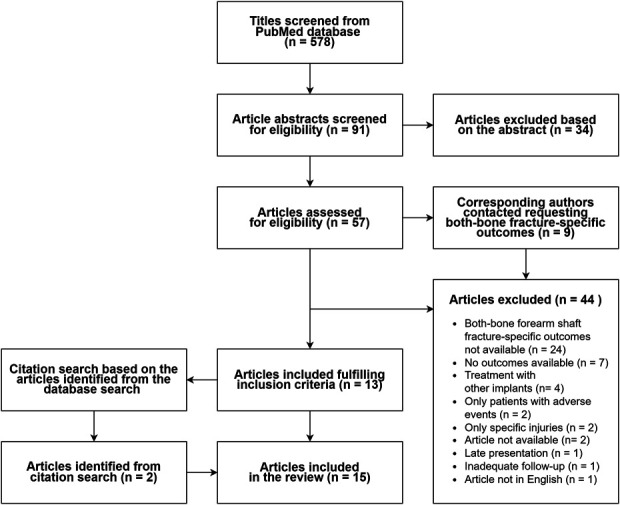
Flow diagram describing the review process

We conducted a comprehensive literature search using the PubMed database. PubMed includes primarily the MEDLINE database and references for entries before they are indexed to MEDLINE. The terms used were Radius AND Ulna, Forearm, Antebrachium, Both Bone, Shaft, Diaphysis, Fracture, and Adult. The complete search syntax is presented in the Appendix. The database search yielded 578 results. In addition, we performed an additional citation search from the studies that were ultimately included in the database search. The whole inclusion process is presented in Figure 1.

Two authors (H.V. [orthopaedic resident] and P.H.N. [hand surgery consultant]) performed the initial title, abstract, and article screening process. Third author (A.S. [orthopaedic consultant]) resolved disagreements between the reviewers. The search was conducted in February 2024.

### Inclusion and Exclusion Criteria

The inclusion and exclusion criteria are elaborated in Table I. If a study reported other types of forearm shaft fractures in addition to BBFFs, we included the patients who had outcomes specific to BBFFs available. If the BBFF-specific data were unavailable, we contacted the corresponding authors requesting specified data. Because of the rarity of BBFF-related publications, we included prospective trials and retrospective case series reporting more than 5 patients. In case of overlapping study populations, the included study was selected according to the following order: (1) prospective studies over retrospective case series and (2) the most recent study.

**TABLE I:** Inclusion and Exclusion Criteria Used in the Systematic Review*

Criteria	Inclusion	Exclusion
Study design	• Randomized controlled trials • Prospective trials • Retrospective case series	• Studies with less than 5 reported patients
Fracture type	• Simultaneous fractures of the shafts of the radius and ulna (both-bone forearm shaft fractures)	• Isolated radial or ulnar shaft fractures • Fracture-dislocations (i.e., Monteggia and Galeazzi fractures)
Injury type	• Acute injuries (operative treatment within 2 wk from the injury)	• Studies reporting only patients with pre-existing specific outcomes (i.e., nonunion or plate removal) • Studies reporting only patients with specific associated injuries (i.e., gunshot wounds)
Treatment	• LCP • LC-DCP • DCP • Point-contact fixator • Modern locking plates (i.e., anatomical plates)	• Intramedullary fixation • External fixation • Tubular plates • Reconstruction plates • Nonoperative treatment
Patients	• Skeletally mature patients (defined by either age or closed epiphyses)	• Skeletally immature patients • Cadavers
Follow-up		• Studies without follow-up until the signs of bony union
Outcomes		• Studies not reporting adverse events or outcomes
Data availability		• The article was unavailable • Specific data on BBFFs treated with compression plating unavailable • Article or direct translation not available in English
Other	• Studies published after 1990	• Overlapping patient populations

* DCP = dynamic compression plate, LC-DCP = limited-contact dynamic compression plate, and LCP = locking compression plate.

### Outcomes and Definitions

The primary outcome was to assess and report the postoperative AEs. We used the definitions of AEs from the original studies and divided them into major and minor categories as follows: Minor AEs resolved during the follow-up and did not require secondary operations. Major AEs needed further operations or persisted. We did not include skin scarring or acute peri-implant fractures because of recent injuries. The secondary outcomes of our review included patient-reported outcome measures (PROMs), range of motion, grip strength, and functional outcomes.

### Data Collection

Two authors (H.V. and P.H.N.) independently collected the data from the studies that met the inclusion criteria, after which a third author (A.S.) resolved disagreements.

We collected all available patient and fracture-related demographics. The collected AEs included nonunion, delayed union, postoperative nerve injuries, superficial and deep surgical site infections, fixation failure, malunion, refracture after plate removal, radioulnar synostosis, complex regional pain syndrome, and other AEs such as nonspecified pain or stiffness. As there were considerable differences in the definitions of AEs and the extent of reporting different outcomes, we recorded information regarding the definitions of specific AEs (e.g., nonunion or delayed union). If there were no remarks regarding a specific AE, we recorded the intel as “not available.”

We collected all available PROMs^21^. Investigator-reported functional outcomes were usually assessed on a 4-step scale, using either the criteria established by Anderson et al.^22^ or Grace and Eversmann^23^ and collected when available (see Appendix Table 1; Appendix Table 2).

### Methodological Quality Assessment

We evaluated the methodological quality of the studies using a modified version of the Coleman Methodology Scoring system (mCMS)^24^, which assesses studies between 0, representing a comprehensively flawed study, and 100, reflecting a study that comprehensively avoids different biases, confounding factors, and the effect of chance (see Appendix Table 3).

Two authors (H.V. and T.A. [hand surgery consultant]) independently performed the mCMS scoring, and a third author (A.S.) resolved disagreements. However, the methodological assessment of the study published by Vasara et al. was only assessed by T.A., who was not the author of the publication. Studies with scores between 85 and 100 were considered high, 70 to 84 good, 55 to 69 fair, and less than 55 poor in quality^25^.

### Statistical Analysis

We performed a pooled analysis of the patients included in the studies. Categorical variables, such as AEs and outcome groups, were pooled to establish a single cohort to calculate the incidences of AEs. We reported the incidences of AEs with 95% confidence intervals (CIs), which were computed using the Wilson score interval. We used the mean values for continuous values as they were available for all studies included. For overall estimation, we calculated means and SDs for continuous variables, using the number of patients as the weighting factor. When calculating the AE incidences, we used the total number of patients as the denominator. We used the statistical program SPSS version 29.0.0 (IBM Corp. released November 17th, 2022) for the statistical analysis. The first author, H.V., performed all statistical analyses.

### Source of Funding

H.V. received research grants from The Finnish Research Foundation for Orthopaedics and Traumatology and the Vappu Uuspää Research Foundation, which are both independent nonprofit research foundations. Other authors did not receive any external funding for this study.

## Results

Fifteen studies met the set inclusion criteria. Among the studies, there were 3 randomized clinical trials and 12 retrospective case series (one with a prospective follow-up)^2,3,9-12,16-19,26-30^. Overall, the studies yielded 944 BBFF patients with a mean age of 38 years (range 12-85). Sixty-eight percent (n = 619) of the patients were male. At least, 27% of the patients had sustained open fractures (data unavailable from 2 studies with 50 patients). The average time to fracture union was 4.2 months (data available from 5 studies with 220 patients). The comprehensive inclusion process is presented in Figure 1, and the baseline characteristics are presented in Table II.

**TABLE II:** Characteristics of the Included Studies in Order of Publication Year*

Study	Year	Country	Study Design	Plate Type	N	Follow-up, mo, Mean ± SD (Range)	Male Sex, N (%)	Age, Mean ± SD (Range)	OTA/AO Type, % (A/B/C)	Open Fracture Grade, % (1/2/3)	Additional Exclusion Criteria	mCMS
Ring et al.^16^	2005	United States	Case series	DCP (n = 33) LC-DCP (n = 8)	41	72 (12-168)	31 (76%)	34 (16-85)	R: 19/55/26† U: 22/39/39	R: 7/2/5† U: 32/5/7	Both fractures type A, follow-up <12 mo, plexus injury	44 (poor)
Wang et al.^27^	2005	Taiwan	Case series	DCP	25	75 ± 39 (16-150)	17 (68%)	41 ± 19	NA	64/24/12	Closed fractures	34 (poor)
Droll et al. ^9^	2007	Canada	Case series with prospective follow-up	LC-DCP	30	65 (24-164)	19 (63%)	44 (18-73)	37/33/30	23/7/13	TBI, pathologic fractures	74 (good)
Behnke et al. ^28^	2012	United States	Case series	LC-DCP DCP	27	17 (12-51)	17 (63%)	32 ± 16 (12-70)	NA	15/7/0	Contralateral injuries	32 (poor)
Iacobellis et al.^29^	2014	Italy	Case series	LCP	17	13 (6-39)	12 (86%)	29 (14-62)	47/29/24	0/12/0	AI, OP, long-term corticosteroid use	53 (poor)
Lee et al.^10^	2014	Korea	Randomized controlled trial	LCP	32	20 (18-65)	22 (69%)	47 (15-82)	44/56/0	9/16/6	Severe comminution, OP, AI	76 (good)
Kim et al.^12^	2015	Korea	Case series	DCP	31	17 (12-40)	20 (65%)	35 ± 17	39/26/35	19/3/13	Follow-up <12 mo	36 (poor)
Marcheix et al.^17^	2016	France	Case series	DCP (n = 91) LC-DCP (n = 40)	131	NA	93 (71%)	40 ± 10	43/37/21	24/8/1	—	37 (poor)
Zhang et al.^30^	2016	China	Randomized controlled trial	LCP	21	23 (12-26)	12 (57%)	38 ± 1	38/24/38	0/0/0	Pathologic and open fractures, AI, OP	64 (fair)
Marchand et al.^18^	2021	United States	Case series	LC-DCP (n = 191) Other (n = 20)	213	11 (6-118)	27 (66%)	40 ± 17 (17-82)	NA	31†	Follow-up <6 mo, distal/proximal fx patterns, segmental bone loss, AI	36 (poor)
Polat et al.^19^	2022	Turkey	Case series	LC-DCP	25	25 (12-48)	138 (65%)	32 (19-67)	44/36/20	24/12/0	Grade III open fractures, TBI	40 (poor)
Saini et al.^31^	2023	India	Randomized controlled trial	DCP	20	NA	24 (80%)	41 ± 12 (20-70)	25/40/35	NA	Grade III open fx, pathologic fx, TBI, AI	34 (poor)
Vasara et al.^2^	2023	Finland	Case series	LCP (n = 157) LC-DCP (n = 16) Other (n = 27)	202	9 ± 15 (1-106)	16 (64%)	39 ± 20 (16-89)	44/42/14	25/16/4	—	47 (poor)
Sahoo et al.^11^	2023	India	Case series	DCP LC-DCP	30	NA	24 (80%)	35 (14-69)	R: 23/6/3† U: 22/4/4	NA	Follow-up <6 mo, contralateral injuries, TBI, AI	30 (poor)
Rust et al.^3^	2024	United States	Case series	LCP Other	99	4 (3-6)	18 (90%)	39 ± 18	NA	32‡	Follow-up <6 mo pathologic fractures	33 (poor)
Total/weighted average	2005-2024				944	14 (1-168)	619 (68%)	38 (12-85)	42/39/19§	23/12/4#		42 (poor)

* AI = Associated injuries of the affected upper extremity (i.e., humeral fractures, proximal, or distal forearm fractures), DCP = dynamic compression plate, Fx = fracture, LC-DCP = limited-contact dynamic compression plate, LCP = locking compression plate, OP = osteoporosis, and TBI = traumatic brain injury.

† The study listed fracture characteristics separately for radius I and ulna (U). Values are not included in the total percentage.

‡ Only the combined incidence of open fractures was available. Values are not included in the total percentage.

§ Data available in 509 patients.

# Data available in 541 patients.

### Adverse Events

There were 224 patients (incidence 24% [CI 21%-27%]) with 251 reported AEs. In total, 142 patients (15% [CI 13-17]) sustained 154 major AEs requiring secondary operations or being persistent. Furthermore, there were 77 patients (8% [CI 7-10]) with 93 minor AEs that resolved during the follow-up (Table III). The most common AEs were postoperative nerve injuries (7% [CI 5-9], n = 64), nonunion (5% [CI 4-6%], n = 45), delayed union (4% [CI 3-6], n = 40), and surgical site infections (4% [CI 3-5%], n = 37). Approximately 66% (n = 54/82) of the nerve injuries affected the sensory system, and the most commonly injured nerve was the superficial radial nerve (Table IV). Six patients experienced a refracture after plate removal. Plate removal (not accounted as an AE) was performed for 58 patients (data available from 486 patients, incidence 12% [CI 9-15]), and therefore, the rate of refracture after plate removal was 10% (CI 5-21). All reported AEs are listed in Table III, and study-specific AEs are reported in Appendix Table 4^2,3,9-12,16-19,26-30^.

**TABLE III:** Adverse Events and Their Incidence Summarized

	N*	Incidence† (%)	Confidence Interval	Data Reported‡ (N)
No. of patients with adverse events	224	24	21-27	944
No. of adverse events	251			
No. of patients with major adverse events	142	15	13-17	
Persistent postoperative nerve injury	15	1.6	1.0-2.6	627
Unspecified postoperative nerve injury	18	1.9	1.2-3.0	627
Nonunion	45	4.8	3.6-6.3	994
Deep surgical site infection	15	1.6	1.0-2.6	882
Fixation failure	16	1.7	1.0-2.7	560
Nonspecified pain or stiffness	14	1.5	0.9-2.5	321
Other hardware complications	10	1.1	0.6-1.9	415
Refracture after plate removal	6	0.6	0.3-1.4	321
Radioulnar synostosis	9	1.0	0.5-1.8	433
Complex regional pain syndrome	3	0.3	0.1-1.0	261
Malunion	2	0.2	0.1-0.8	402
Compartment syndrome	1	0.1	0.0-0.6	301
No. of major adverse events	154			
No. of patients with minor adverse events	77	8	7-10	
Delayed union	40	4.2	3.1-5.7	422
Transient postoperative nerve injury	31	3.2	2.3-4.6	627
Superficial surgical site infection	22	2.3	1.5-3.5	460
No. of minor adverse events	93			

* The sum of the specific adverse events does not add up with the total number because of inconsistencies in the reporting in 2 publications.

† The total number of patients (n = 944) was used as denominator.

‡ The number of patients in the studies that have mentioned the specific adverse event.

**TABLE IV:** Nerve Injuries Categorized by Their Anatomical Location and Functional Deficit*

	N	Proportion of Nerve Injuries (%)
Motor palsy	28	34
Posterior interosseus nerve	10	12
Anterior interosseus nerve	6	7
Median nerve, main branch	4	5
Ulnar nerve	3	4
Unspecified motor palsy	4	5
Sensory paresthesia	54	66
Superficial radial nerve	14	17
Median nerve	6	7
Ulnar nerve	9	11
Lateral cutaneous nerve of the forearm	1	1
Unspecified sensory paresthesia	24	29
Total no. of nerve injuries specified	82	100

* The data were available from 10 studies with total of 627 patients, 64 of which had postoperative nerve injuries. A single patient might have had injuries in multiple nerves.

### Patient-Reported Outcome Measures and Functional Outcomes

All available PROMs and range of motion measurements are presented in Table V. The Disabilities of the Arm, Shoulder, and Hand (DASH) questionnaire score^21^ was reported in 5 studies with 135 patients. The weighted mean DASH score in these studies was 12.5 (SD, range 0-61) assessed at a mean of 28 months (range 6-164 months).

**TABLE V:** Summary of the Studies Reporting Patient-Reported Outcomes or Range of Motion Measurements*

Authors	DASH, Mean ± SD (Range)	Forearm Rotation Arc° Mean ± SD (Range)	Pronation° Mean ± SD (range) [% from Contralateral]	Supination° Mean ± SD (Range) [% from Contralateral]	Other Measurements Mean ± SD (Range) [% from Contralateral]	Time When Assessed, mo, Mean ± SD (Range)
Droll et al.	18.3 ± 18 (0-61)	162	80 ± 18 [91%]	82 ± 18 [90%]	SF-36 PCS 49 ± 10 (32-64) SF-36 MCS 47 ± 11 (20-64) Wrist flexion 57° ± 18° [82%] Wrist extension 61° ± 13° [93%] Grip strength [75%]	64.8 (24-164)
Behnke et al.	NA	161 ± 33 (40-180)	82 ± 17 (30-90)	79 ± 22 (10-90)		16.5 (12-51)
Iaccobellis et al.	14.3 ± 11.5 (5-46.7)	173 ± 7	NA	NA	Elbow ROM deficit 4° (0-20)	13 (6-39)
Lee et al.	15 ± 3	159 ± 5	NA	NA	Patient satisfaction 2.8§	20 (18-65)
Kim et al.	7.1 (0-19.2)	174‡	89 (60-90)	85 (70-90)		16.8 (12-40)
Marcheix et al.	NA	146‡	76	73		NA
Polat et al.	8.4 (0-30)	160‡	80 (60-90)	80 (60-80)	Grip strength mean 39 kg	24.8 (12-48)
Saini et al.	NA	147‡	70	77	Elbow ROM 118°	NA
Sahoo et al.	6 ± 6.6† (0-25)	131‡	57 [69%]	74 [82%]	PRWE 7±4.5 Wrist flexion 53° ± 8° [53%] Wrist extension 48° ± 11° [70%] Elbow ROM 139 ± 6 [94%] Grip strength 35 kg [81%]	NA
Weighted mean	12.5 (0-61)	153	76	77		

* DASH = Disabilities of the Arm, Shoulder, and Hand, MCS = mental component score, PCS = physical component score, PRWE = patient-reported wrist evaluation, ROM = range of motion, and SF-36 = Short Form-36 Health questionnaire. Scores range from 0 to 100, and the mean score of 50 represents the normative value. In DASH, quick DASH, and PRWE, the score 0 represents no disability, whereas 100 represents the worst functional score.

† Reported as QuickDASH.

‡ Not reported separately in the article, calculated as the sum of the pronation and supination.

§ Reported as a scale from 1 to 4 (insufficient = 1, satisfactory = 2, good = 3, or very good = 4).

The forearm rotation arc was available in 9 studies with 343 patients. The average rotation arc was 153°. Separate pronation and supination were available in 7 studies with 294 patients. The average pronation was 76°, whereas the average supination was 77°.

Anderson et al. or Grace and Eversmann's investigator-reported functional criteria were available in 10 studies. Five studies (n = 135) reported the Anderson criteria: 61% (CI 52-69) of the patients achieved excellent outcomes preserving at least 75% of the forearm rotation. Furthermore, 22% (CI 16-30) of the patients had satisfactory and 10% (CI 6-16) unsatisfactory outcomes. Seven percent of the patients (CI 4-13) were defined as failure, not achieving union (see Appendix Table 1). Five studies (n = 123) reported the Grace and Eversmann criteria 63% (CI 55-71), and 17% (CI 11-25) of patients achieved excellent and good outcomes, respectively, preserving at least 80% of the normal forearm range of motion. Furthermore, 14% (CI 9-21) were classified as acceptable and 6% (CI 3-11) as unacceptable (see Appendix Table 2)

### Methodological Quality

According to the modified mCMS, there were 2 good-, 1 fair-, and 12 poor-quality studies. The average nonweighted average score was 45 (range 30-76) (Table I).

## Discussion

According to our systematic review, the average AE incidence after compression plate fixation of BBFFs was 24%. The most frequent AEs were postoperative nerve damage (7%) and nonunion (5%). According to available PROMs and investigator-reported functional outcomes, the average patient seems to recover well, with only minor disabilities in their activities of daily living. The overall quality of the current literature is poor, with only few available prospective studies. This is the first systematic review conducted on AEs after BBFFs treated with compression plating in adults.

The incidence of AEs varied greatly in the studies included in the review, with the lowest being 5%^19^ and the highest being 45%^3^. We believe this is explained mainly by 4 factors. First, there was considerable variance in the definitions of the AEs, especially in the case of nonunion and delayed union^2,3^. Second, in some studies, there might be underreporting of minor AEs, such as superficial infections, delayed union, and transient nerve injuries. Although underreporting is difficult to showcase, for example, nerve injuries were mentioned in only 10 of the 15 studies included^2,3,9,11,16-18,27,28,31^. Third, the fracture characteristics and associated injuries were different. It is previously established that open fractures and comminuted B- and C-type fractures pose a higher risk of AEs^2^. Finally, institutional factors, such as surgeon experience and hospital protocols, are likely to have an impact on the incidence of AEs^2,28^.

According to our systematic review, the functional outcomes after BBFFs are generally satisfactory. The mean DASH score was 12.5, indicating comparable disability with the general population^32,33^. However, DASH scores were available for a few patients, and some studies did not report the DASH scores from patients who sustained nonunion^28^. The 4-step functional outcome assessments by Anderson et al. and Grace and Eversmann were the most used outcome measurement tools^7,10,11,16,17,26-30^. These assessments account only for the range of motion and fracture union without considering functionality in daily activities and grip strength. Overall, studies reporting outcomes using PROMs, such as DASH, are evidently needed.

The overall quality of the published literature is poor and consists of considerable methodological flaws. There were only 4 studies with prospective follow-up, and currently, there are no high-quality studies reporting outcomes after BBFFs. In addition, no ongoing studies are registered for ClinicalTrials.gov assessing adult BBFFs or isolated radius fractures and only a single ongoing study assessing ulnar shaft fractures. At the moment, the treatment is based on poor evidence, expert opinions, and institutional protocols, and thus, there is a clear clinical need for high-quality clinical studies to enhance patient care.

### Limitations

As described above, the most considerable limitation of this review arises because of the poor methodological quality of the available studies. There was substantial heterogeneity between the studies, which reduces the generalizability. However, the results presented in this review article represent the best possible estimate of the outcomes of the available literature. Second, we also included older implants such as DCP. Although they possess a higher risk of nonunion and delayed union, excluding studies with older-generation implants would have drastically reduced the number of studies available. In addition, as our review indicates DCPs are still commonly used in some institutions and including them reflects the reality of current BBFF treatment internationally. Overall, the limitations discussed above need to be taken into account before applying our results for individual practices and, for example, using them for informing patients.

## Conclusions

Patients with both bone forearm shaft fractures should be informed of the moderately high incidence of AEs (24%). According to the studies reporting PROMs, the average functional outcomes are good, although minor disability typically persists to some extent. There is an obvious need for high-quality prospective trials assessing the outcomes and treatment of BBFFs in adults to steer the treatment of forearm fractures to a more evidence-based direction.

## Appendix

Supporting material provided by the author is posted with the online version of this article as a data supplement at jbjs.org (http://links.lww.com/JBJSOA/A705). This content has not been copyedited or verified.
